# Supplementary material for: Multiresistance states in ferro- and antiferroelectric trilayer boron nitride
Source: Nat Commun. 2024 Jan 5;15:295. doi: 10.1038/s41467-023-44617-w (PMC10766609; doi:10.1038/s41467-023-44617-w)
Supplement: Supplementary file 1 — Supplementary Information [file 41467_2023_44617_MOESM1_ESM.pdf]

Supplementary Information

Multiresistance States in Ferro- and Antiferroelectric Trilayer Boron Nitride

Ming Lv[1†], Jiulong Wang[2†], Ming Tian[3†], Neng Wan[3*], Wenyi Tong[2*],Chungang Duan[2,4], Jiamin Xue[1*]

[1]School of Physical Science and Technology, ShanghaiTech University, Shanghai, 201210, China

[2]Key Laboratory of Polar Materials and Devices (MOE), Ministry of Education, Department of Electronics, East China Normal University, Shanghai, 200241, China

[3]Key laboratory of MEMS of Ministry of Education, School of Integrated Circuits, Southeast University, Nanjing 210096, China

[4]Collaborative Innovation Center of Extreme Optics, Shanxi University, Taiyuan, Shanxi 030006, China

ABC	BAB	CBA	ABA
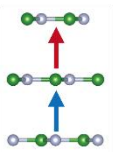	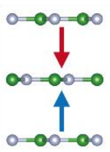	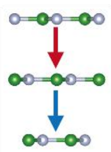	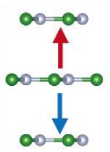
↑↑	↓↑	↓↓	↑↓

Table. S1 Polarization notation definitions. Each column represents three equivalent notations for a particular stacking and polarization state.

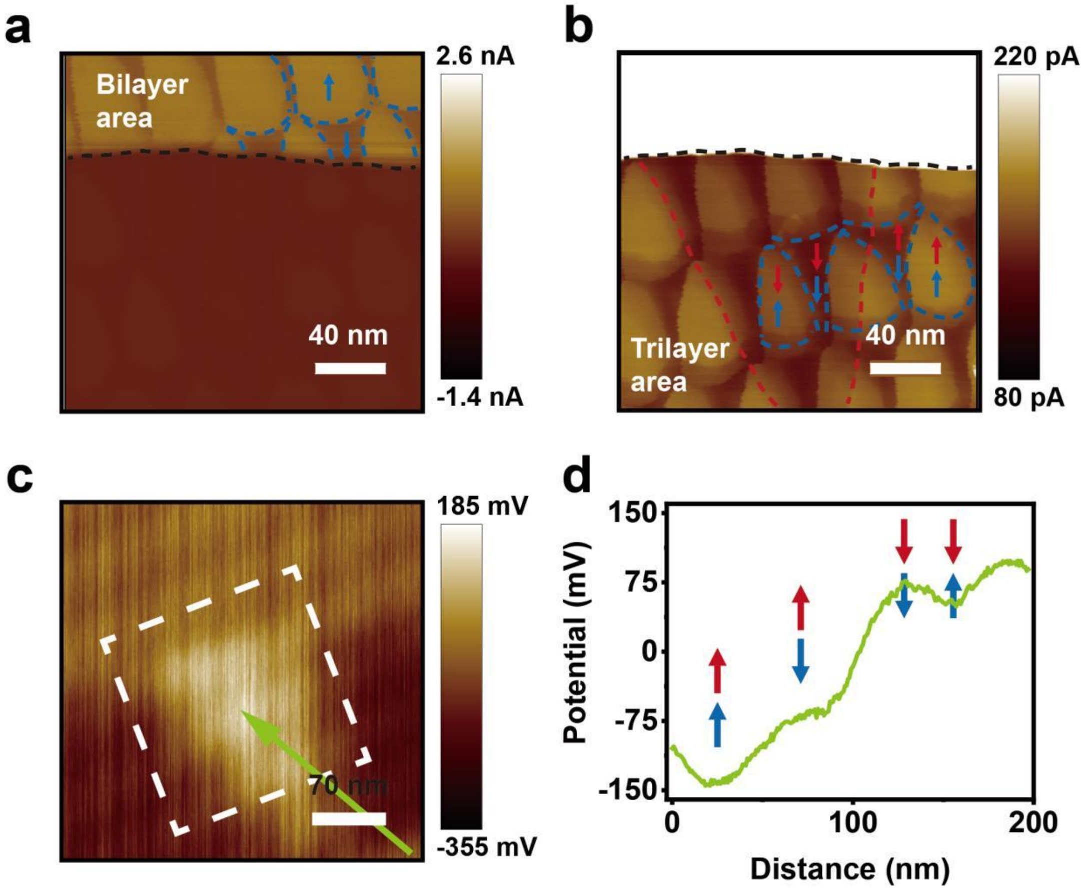

Fig. S1 CAFM mapping near a step between the bilayer and trilayer BN. a and **b** CAFM images around the step at 100 mV sample bias voltage. These two images are from the same scan but displayed with different color scales, since the bilayer and trilayer areas have an order of magnitude difference in current. The blue dashed lines outline the domains from the bottom two layers of BN, and the red dashed lines outline one domain from the top two layers of BN. **c** Corresponding KPFM image around the step with a larger scanning area. The white dashed box corresponds to the area shown in **a** and **b**. **d** Profiles of the potential along the green arrow in **c**.

We use a step edge between the bilayer and trilayer BN to identify the origin of the polarization in different domains. As shown in Figs. S1a and b, the bottom (top) part is the trilayer (bilayer) area. It is evident that the smaller triangular domains come from the bottom interface while the larger ones come from the top. Then the sample bias is swept. The expansion or shrinkage of the triangular domains in response to the sample bias can unambiguously tell the polarization of each domain, e.g. if a domain shrinks when the sample bias is increased, then its polarization is pointing to sample substrate (for more details, see *Adv. Mater.* **34**, 2203990 (2022)). All the polarization states can be fixed this way and are labeled in Figs. S1a and b.

Then SKPM can be used to map out the surface potential. Fig. S1c shows such a scan, with the white dashed box corresponding to the area in Figs. S1a and b. It is a zoomed-out scan

compared with Figs. S1a and b to cover a larger area due to the lower resolution of SKPM. A line cut along the green arrow is shown in Fig. S1d with the polarization combinations assigned. It can be seen that the ↑↑ has the lowest potential while ↓↓ the highest. The second largest potential comes from the ↓↑ domain and its value is quite close to that of the ↓↓ domain. This is understandable since the flip of the bottom polarization that is farther away from the SKPM tip produces a smaller change in potential. This correspondence between the surface potential and polarization can be used to identify other domains. We note that the resistance order of the polarization states at low sample bias voltage can also be used as shown in the discussion about Fig. 3 in the main text. These two methods corroborate each other to confirm the assignment of polarization state to each domain.

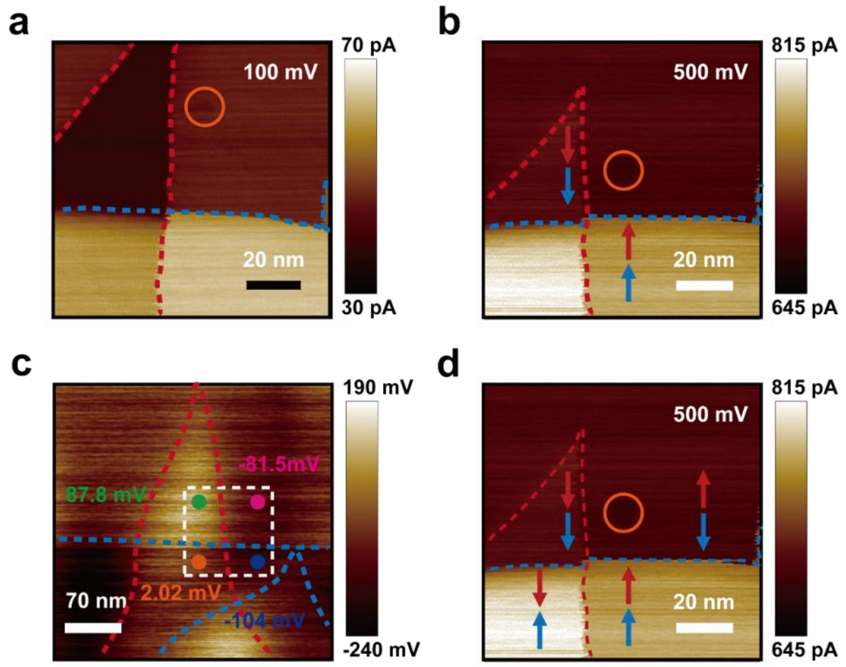

Fig. S2 An example of using the SKPM to identify polarization states. a and **b** CAFM images at 100 mV and 500 mV sample bias. The orange circles in **a** and **b** highlight a defect to show the thermal drift during scan. The ferroelectric domains can be easily identified from the change of their shapes, and are marked out in **b**. The triangular domain in the upper left corner has an observable size reduction, while the domain in the lower right corner appears to be the same from **a** to **b**. This is due to the drift effect. With the defect as a reference, we can see that its horizontal domain wall has moved upwards. **c** KPFM image around the same area with a zoomed-out scan. The numbers are averaged surface potentials at each domain. The white dashed box corresponds to the area in **a** and **b**. **d** A CAFM image with all the polarization states identified.

Fig. S2 shows an example of polarization identification with the help of KPFM. Figs. S2a and b are CAFM images at 100 mV and 500 mV, respectively. The ferroelectric domains are easily identified from their size change, e.g. the triangular domain on the top left side has its polarization pointing down since it shrinks. On the other hand, the antiferroeletric domains need more information to determine their specific states. A KPFM scan around this area maps

out the surface potentials as shown in Fig. S2c, with the white dashed box corresponds to the area in Figs. S2a and b. Note that in the KPFM scan no sample bias was applied so the domain sizes and shapes are different from those in Figs. S2a and b. According to the information from Fig. S1, all the polarization states can now be fixed as shown in Fig. S2d.

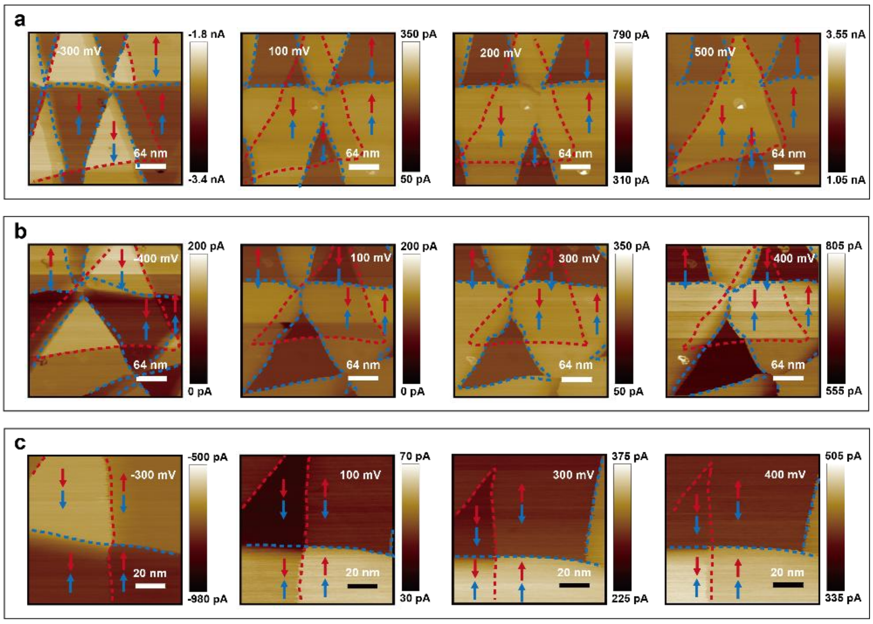

Fig. S3 Evolution of the current values in different samples. a-c CAFM images with different sample bias in three samples. It can be seen that at low bias, the current order from largest to smallest is ↑↑, ↓↑, ↓↓, and ↑↓.

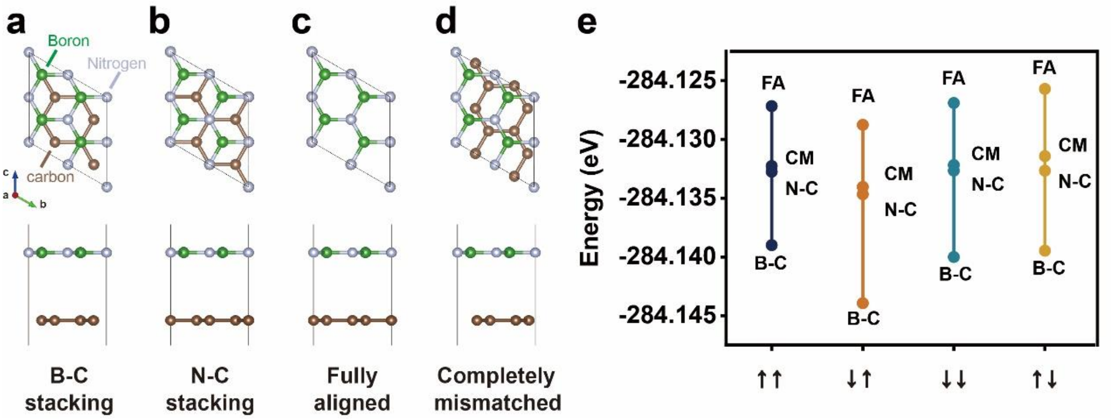

Fig. S4 Different stacking configurations and their corresponding energies. a-d Top and side views of the four stacking types at the BN and graphite interface considered in this work, which are B-C stacking, N-C stacking, fully aligned (FA) and competently mismatched (CM), respectively. **e** Calculated stacking energies of these four types for the four different polarization combinations. B-C stacking always has the lowest energy.

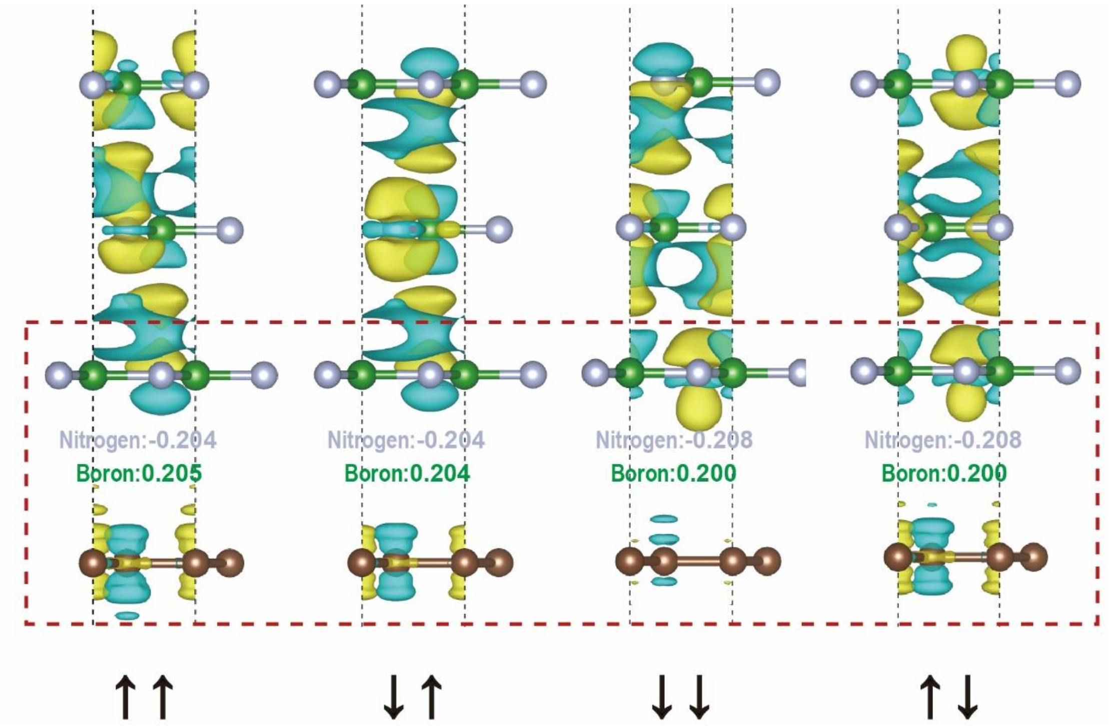

Fig. S5. Differential charge density distributions where yellow and blue isosurfaces indicate electron accumulation and depletion after layer stacking, respectively. The Hirshfield charges for the BN layer neighboring to graphene are labeled.

The ↑↑ and ↓↑ polarization states share the same ↑ polarization formed by the lower two BN layers. As a result, their electronic environments at the BN/graphene interface are similar yet very different from those of the ↓↓ and ↑↓ states. As displayed in Fig. S5, the N atoms of the↑↑ and ↓↑ states lose electrons (blue isosurfaces) on their sides facing graphene. In contrast, the N atoms the ↓↓ and ↑↓ group accumulate electrons (yellow isosurfaces). We then performed the Hirshfeld charge analysis of the bottom BN layer. For the ↑↑ and ↓↑ polarization states, B/N atoms have similar charges of ~ 0.205e/−0.204e, making the BN layer almost electroneutral. On the other hand, the bottom BN layer in the ↓↓ and ↑↓ polarization states carries charge of ~ −0.008e per unit cell.

Our differential charge density distributions, in accompany with Hirshfeld charge analysis, clearly demonstrate that due to the across-layer effect, the impact of graphene substrate on the trilayer BN differs depending on the polarization state of the lower two layers of BN.

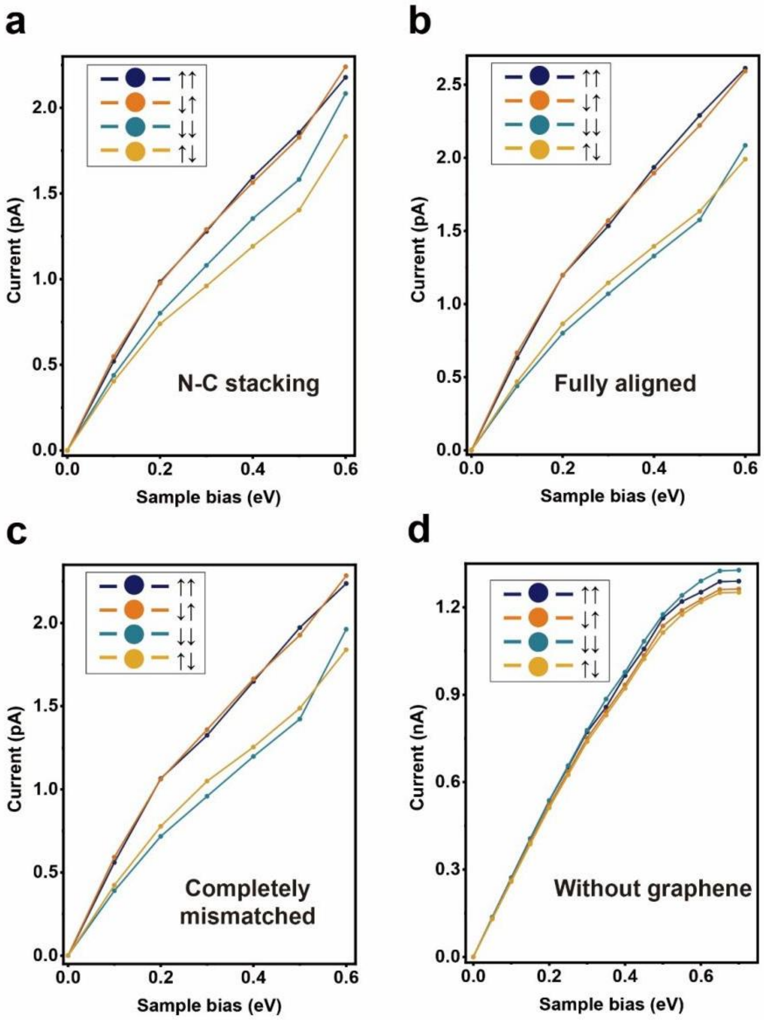

Fig. S6. Calculated *I-V* curves of different stacking configurations. a *I-V* curves of N-C stacking. **b** *I-V* curves of B-C stacking. **c** *I-V* curves of the completely mismatched configuration. **d** *I-V* curves without the graphene layer.

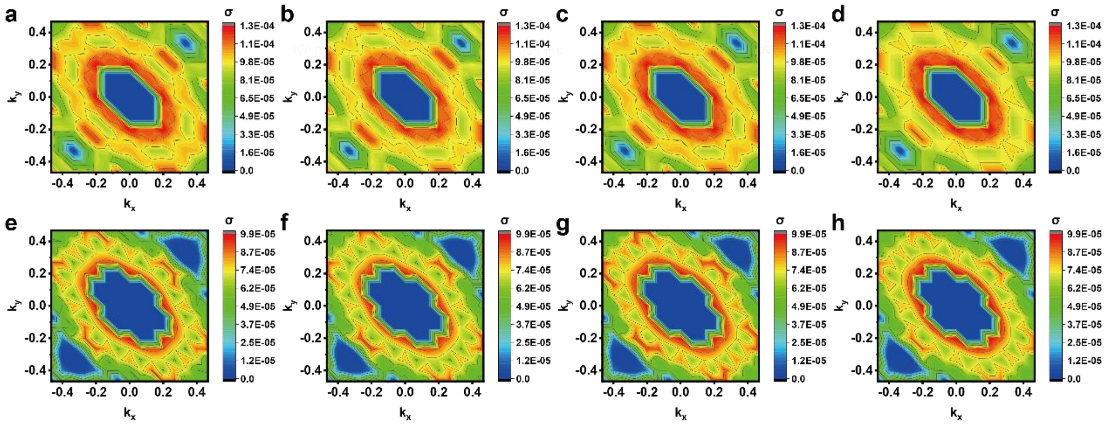

Fig. S7 $k_{||}$-resolved transmissions in 2D Brillouin zone at the Fermi energy of trilayer BN without graphene. a-d ↑↑, ↓↑, ↓↓ and ↑↓ polarization states under 0 V bias. **e-h** The same polarization states under 0.6 V bias.

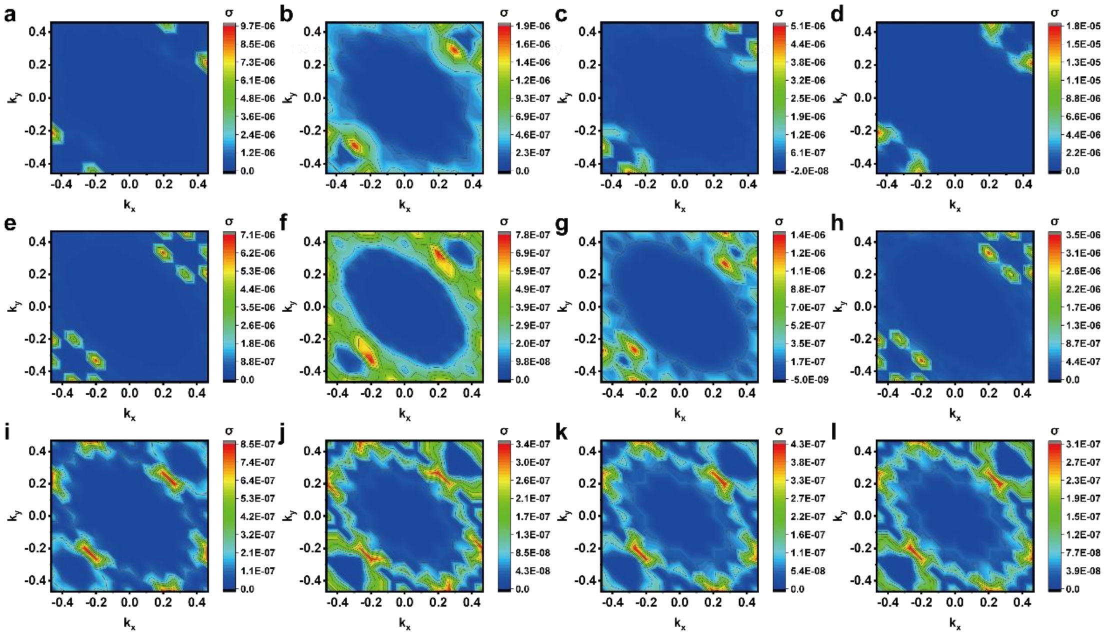

Fig. S8 $k_{||}$-resolved transmissions in 2D Brillouin zone at the Fermi energy of trilayer BN with graphene. a-d ↑↑, ↓↑, ↓↓ and ↑↓ polarization states under 0 V bias. **e-h** The same polarization states under 0.1 V bias. **i-l** The same polarization states under 0.6 V bias.

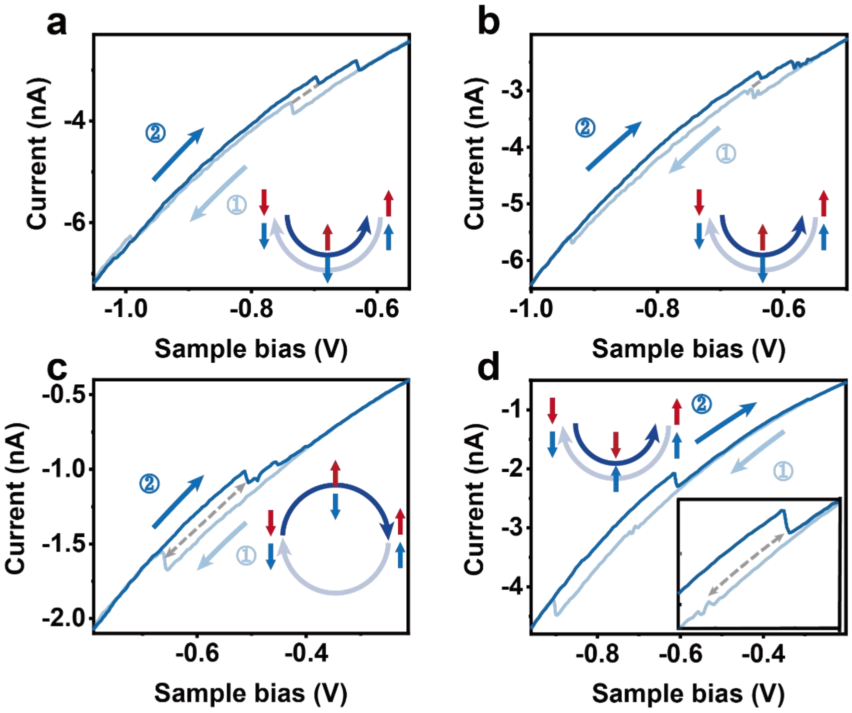

Fig. S9 Other layer-by-layer switching data of the ferroelectricity domains. **a, b** More examples showing that the majority switching route flips the bottom polarization first in the forward scan. **c** A rare case that has no intermediate state in the forward scan. **d** A rare case that flips the top polarization first in the forward scan.
